# Supplementary material for: Mixture of personality improved spiking actor network for efficient multi-agent cooperation
Source: Front Neurosci. 2023 Jul 6;17:1219405. doi: 10.3389/fnins.2023.1219405 (PMC10361619; doi:10.3389/fnins.2023.1219405)
Supplement: Supplementary file 1 [file Data_Sheet_1.PDF]

frontiers

Supplementary Material

1 SUPPLEMENTARY TABLES AND FIGURES

1.1 Figures

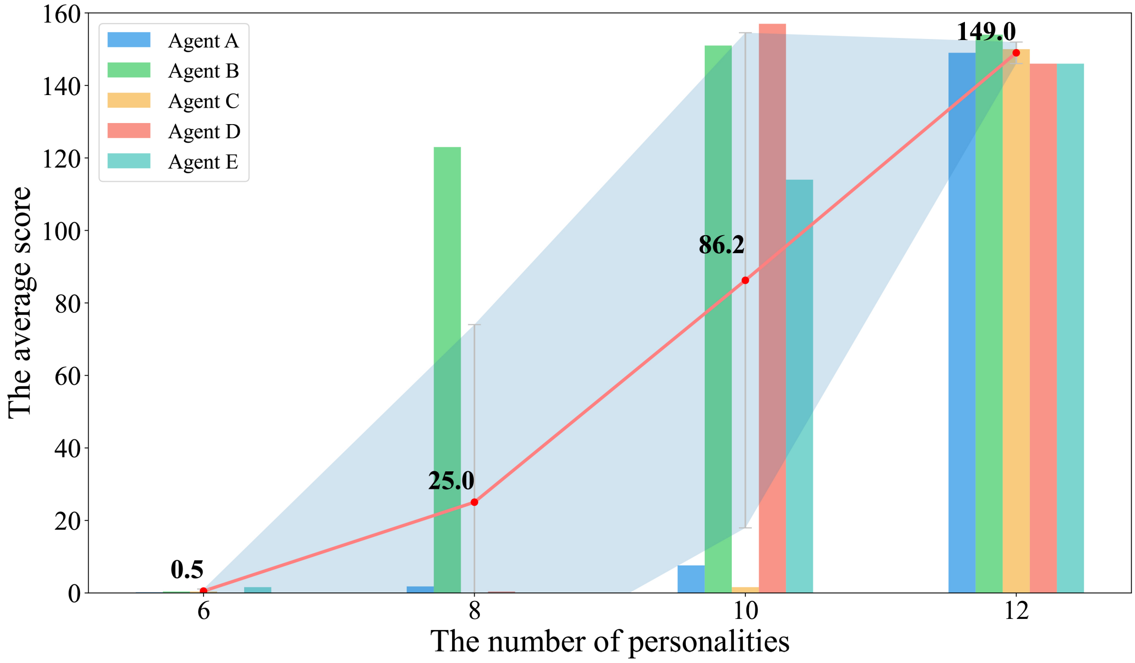

Figure S1. This diagram indicates the detailed scores for different personality numbers (6, 8, 10, 12). A-E denotes different agent with different random seeds. In our method, the score drastically improves as the number of personalities increases.

1.2 Tables

Table S1. Mean rewards for all methods.

Methods	A	B	C	D	E	avg
**DNN baseline**	**192**	**202**	**202**	167	**192**	**191.0 (±14.32)**
**SAN baseline**	136	154	164	**173**	12	127.8 (±66.18)
**MoP-SAN (ours)**	149	147	146	150	154	149.2 (±3.32)

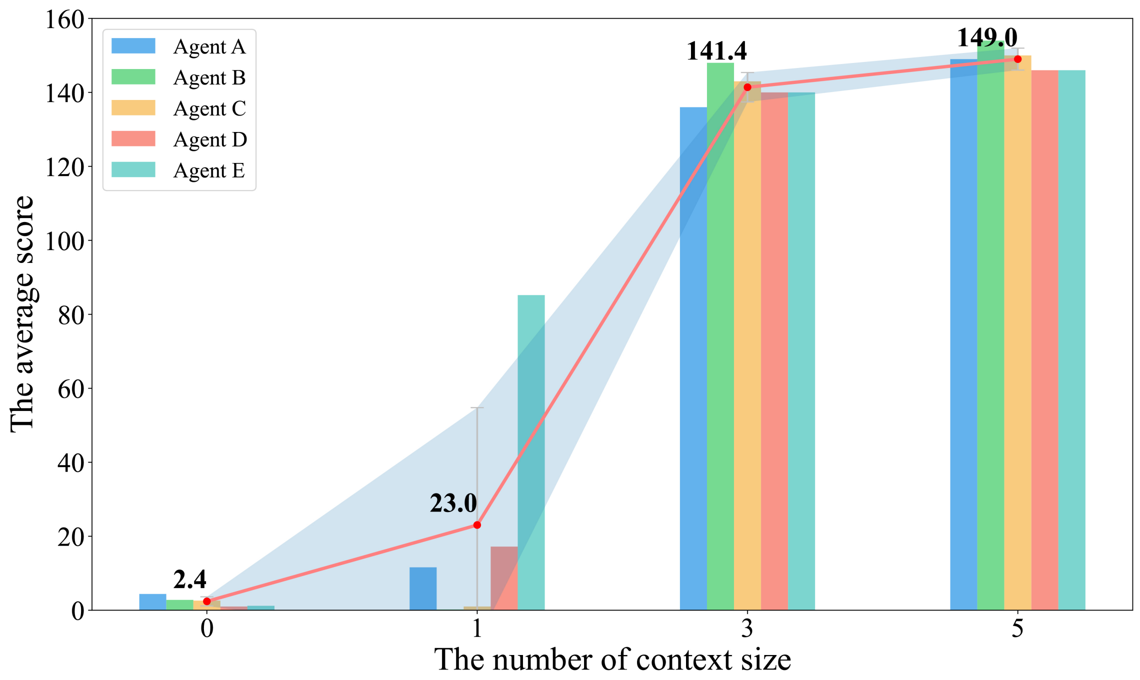

Figure S2. This diagram shows the detailed scores for different context sizes (0, 1, 3, 5). A-E denotes different agents with different random seeds. The score improves as the number of contexts increases in our method.

Table S2. Mean value of overall generalization rewards in the generalization phase.

Methods	A	B	C	D	E	avg
**DNN baseline**	37.5	12	53	27.5	79	41.8 (±25.59)
**SAN baseline**	115.7	111.9	102	85.6	143.8	112.2 (±19.11)
**MoP-SAN (ours)**	**143**	**149.3**	**152**	**158.8**	**157**	**152.0 (±6.32)**

Table S3. The mean score of our method w/o transformer context encoder and our method.

Methods	A	B	C	D	E	avg
**ours w/o context encoder**	4.4	2.8	2.6	1	1.2	2.4 (±1.38)
**ours**	**149**	**154**	**150**	**146**	**146**	**149 (±3.32)**

Table S4. The mean generalization score of our method w/o DPP module and our method.

Methods	A	B	C	D	E	avg
**ours w/o DPP module**	124	131	129	119.5	153.7	131.4 (±13.22)
**ours**	**143**	**149.3**	**152**	**158.8**	**157**	**152.0 (±6.32)**
